# Prospective sarcopenia outcomes associated with physical performance in individuals aged 55 years and over in Malaysia

**DOI:** 10.3389/fpubh.2023.1226642

**Published:** 2023-10-12

**Authors:** Intan Meinar Megasari, Sumaiyah Mat, Devinder Kaur Ajit Singh, Maw Pin Tan

**Affiliations:** ^1^Centre for Healthy Ageing and Wellness, Physiotherapy program, Faculty of Health Sciences, Universiti Kebangsaan Malaysia, Kuala Lumpur, Malaysia; ^2^Division of Geriatric Medicine, Department of Medicine, Faculty of Medicine, University of Malaya, Kuala Lumpur, Malaysia; ^3^Department of Medical Sciences, School of Medical and Life Sciences, Sunway University, Bandar Sunway, Malaysia

**Keywords:** sarcopenia, physical performance test, SARC-F, older adults, cut-off value

## Abstract

**Background:**

While the potential of physical performance tests as screening tools for sarcopenia is evident, limited information on relevant reference values for sarcopenia detection. In this study, we aimed to establish the prospective relationship between physical performance tests, including time up and go (TUG), functional reach (FR), gait speed (GS), and hand grip strength (HGS) with five-year sarcopenia risk and to determine suitable cut-off values for screening activities.

**Method:**

This was a prospective study utilizing data from the Malaysian Elders Longitudinal Research (MELoR) study, which involved community-dwelling older adults aged 55 years and above at recruitment. Baseline (2013–2015) and wave 3 (2019) data were analyzed. Sarcopenia risk was determined using the strength, assistance walking, rising from a chair, climbing stairs, and falls (SARC-F) tool, with SARC-F ≥ 4 indicating sarcopenia. Baseline physical performance test scores were dichotomized using ROC-determined cut-offs.

**Result:**

Data were available from 774 participants with mean age of 68.13 (SD = 7.13) years, 56.7% women. Cut-offs values for reduced GS, TUG, FR, and HGS were: <0.7 m/s (72.9% sensitivity and 53% specificity), >11.5 s (74.2%; 57.2%), <22.5 cm (73%; 54.2%) and HGS male <22 kg (70.0%; 26.7%) and female <17 kg (70.0%; 20.3%) respectively. Except for FR = 1.76 (1.01–3.06), GS = 2.29 (1.29–4.06), and TUG = 1.77 (1.00–3.13) were associated with increased sarcopenia risk after adjustments for baseline demographics and sarcopenia.

**Conclusion:**

The defined cut-off values may be useful for the early detection of five-year sarcopenia risk in clinical and community settings. Despite HGS being a commonly used test to assess strength capacity in older adults, we advocate alternative strength measures, such as the sit-to-stand test, to be included in the assessment. Future studies should incorporate imaging modalities in the classification of sarcopenia to corroborate current study findings.

## 1. Introduction

Sarcopenia has been reported in up to 29 percent of community-dwelling older persons worldwide, though prevalence varies according to definitions, study setting, and population selection (1). The prevalence of sarcopenia is higher among nursing home residents with a 33 percent prevalence previously reported (2, 3). Among older persons with disabilities or those who receive rehabilitation, the prevalence rises to 78 percent (4, 5). The highest prevalence of sarcopenia has been reported in low- and middle-income countries (LMICs) (6) with a 7 to 44 percent prevalence reported in Malaysia (7).

Sarcopenia negatively impacts afflicted older adults’ quality of life. The reduction in muscle quality and quantity leads to decreased mobility, and in the longer term, increased dependency. Moreover, sarcopenia has been recognized as an independent condition by the World Health Organization through its listing in the International Classification of Diseases ICD-10 suggesting the need for early prevention and management strategies (8). Loss of functional capacity in older adults manifests as greater difficulties in completing basic tasks such as walking at a regular pace, loss of muscle strength, and mobility impairments. Poor physical performance is associated with sarcopenia (9–11), frailty (12, 13), and cognitive impairment (14–16).

While sarcopenia is a common age-related issue with calls for opportunistic screening in the primary care setting (17), the availability of published cut-off scores for physical performance tests commonly used to predict sarcopenia remains limited. Further, healthcare practitioners currently lack the knowledge or training necessary to identify and manage physical capacity losses as people age (18). Previous studies have utilized short physical performance battery (SPPB), hand grip strength (HGS), and timed up-and-go TUG tests to predict the risk of sarcopenia (11, 19). Poor physical performance in older persons is also related to adverse outcomes in older persons such as falls (12, 20) disability (21) and poor quality of life (22).

In the Asian population, physical performance test cut-off values are largely determined from published studies conducted in the East Asian population (23). Further research from other parts of the continent is needed. In addition, previous studies have addressed cross-sectional detection of the presence of sarcopenia rather than prediction of future risk of sarcopenia. Thus, in this study, we sought to evaluate and establish the predictive ability of the prospective relationship between physical performance tests, including time up and go (TUG), functional reach (FR), gait speed (GS), and hand grip strength (HGS) with five-year sarcopenia for adults aged 55 years and over in Malaysia, using newly established cut-off values. We formulated a hypothesis that the physical performance tests (HGS, TUG, GS, and FR) could accurately predict the risk of sarcopenia after 5-years of follow-up.

## 2. Materials and methods

### 2.1. Study design and data source

This prospective observational study utilized baseline (2013 to 2015) and wave 3 follow-up (2019) data from the Malaysian Elders and Longitudinal Research (MELoR) study. The MELoR study is now the Transforming Cognitive Frailty to Later Life Self-sufficiency (AGELESS) study, which was funded by the Ministry of Higher Education Malaysia Long Term Research Grant Scheme (LR005-2019) LRGS/1/2019/UM//1/1.

### 2.2. Study population

**Figure 1:**
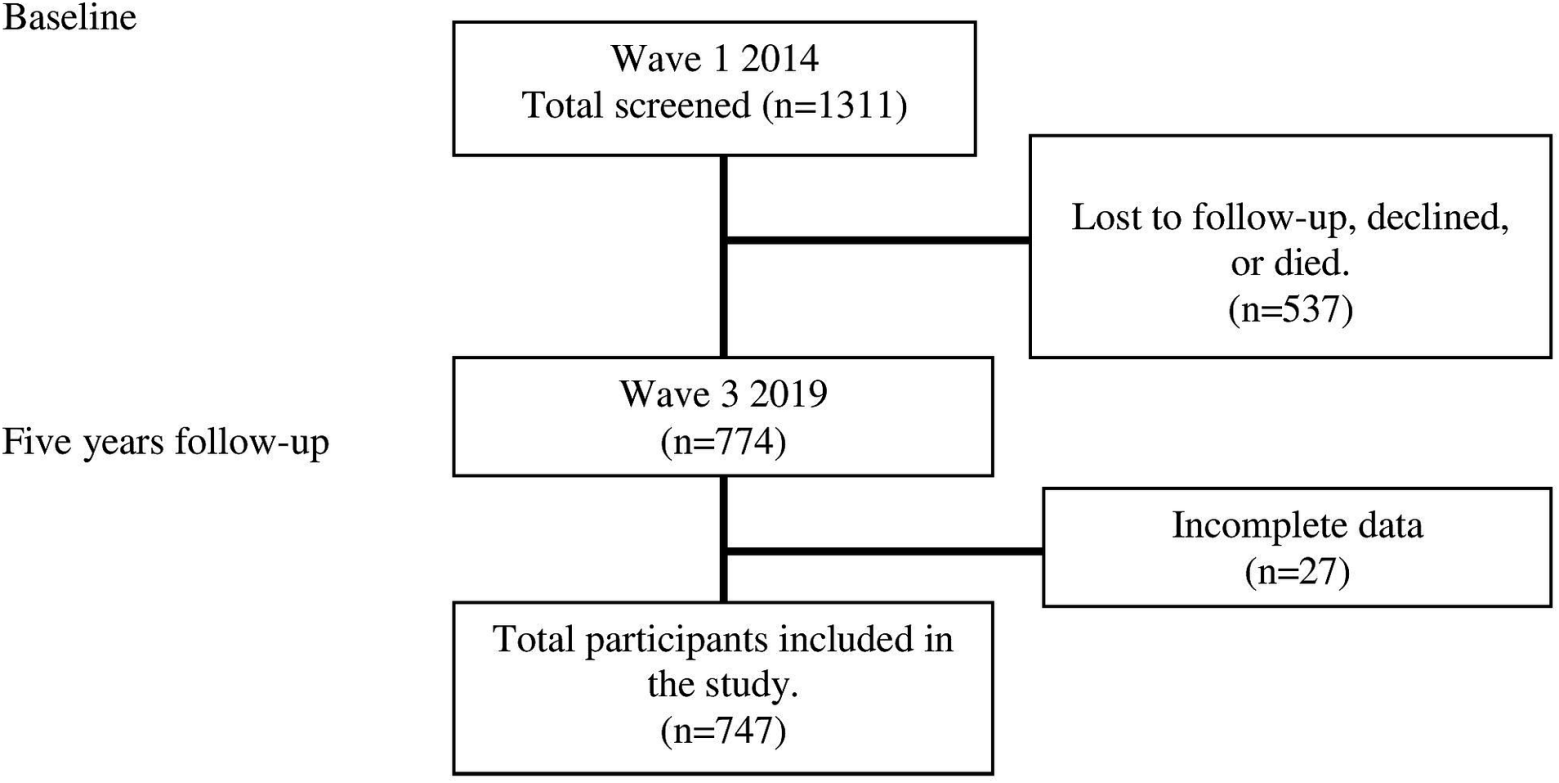
Flowchat of participant recruitment and follow-up.

The study population comprised community-dwelling individuals aged 55 years and above identified from the electoral rolls of three neighboring parliamentary constituencies using simple stratified sampling. Further details on recruitment strategies for the MELoR study have been published elsewhere (24). Baseline measurements of physical performance and potential influencing factors were utilized in this study. A total of 1,311 participants (n = 1,311) were initially collected for this study at the baseline. Following a 5-year follow-up period, 537 participants were lost to follow-up, declined or died. Out of the 774 data initially managed for follow-up, 21 were found to be incomplete. We collected a total of 747 complete data entries for analysis. Telephone follow-up was conducted in 2019 by trained interviewers. Sarcopenia outcomes were obtained from the telephone follow-up interviews (Figure 1).

### 2.3. Baseline assessments

Figure 1 depicts a flowchart of participants and recruitment. The socio-demographic data of age, gender, marital status, medical history, comorbidities, and anthropometric data were collected at baseline (*n* = 1,311). Weight was measured using a digital scale (TANITA type TBF-400). Height was measured using a calibrated standing stadiometer into the nearest centimeter. The body mass index (BMI) was then calculated by weight divided by the body weight in kilograms with the height in meters (kg/m2). Waist and hip circumference were obtained in the standing position using a measuring tape and the waist-to-hip ratio was calculated by dividing the waist measurements with the hip measurements. Older adults aged 55 years and above, residing in the community, demonstrated the ability to stand independently for at least 1 min without any support, walk 7 meters, and have the capability to get in and out of a chair with or without an assistive device were included in the study. However, older adults who were unable to comprehend and follow the instructions for the physical performance tests, those experiencing acute illnesses, and individuals taking medications that could potentially impact their balance during the assessment were excluded from the study.

### 2.4. Sarcopenia screening tools

In this study, Sarcopenia was determined during telephone follow-up using the SARC-F tool, which consists of the five questions: Strength (S), Assistance walking (A), Rising from a chair (R), Climbing stairs (C), and Falls (F), rated from “not at all” to “extremely difficult” on a scale of 0 to 2. The suggested cut-off for the presence of sarcopenia is four points out of the maximal total score of 10 (25, 26). SARC-F scores were determined both at baseline and at five-year follow-up for all participants. SARC-F at baseline involved the substitution of sex-specific lowest quintile for hand grip strength for strength, as the question was not available for the baseline questionnaire.

### 2.5. Physical performance tests

#### 2.5.1. Gait speed

A 10-meter walking path (2-meter acceleration, 6-meter walk, and 2-meter deceleration) was used in this study. The participant was asked to walk using their casual walking speed. The stopwatch was started when the foot first crossed the marked acceleration point and stopped when the foot crossed the first marked deceleration point. The time taken was recorded in seconds. The test was then repeated and the mean of two trials was taken as the result (27).

#### 2.5.2. Time up and go

To conduct the TUG test, a wooden solid chair with its seat at a height of 46 cm above the ground was used. The participant was given verbal instruction prior to the test. The stopwatch is started as soon as the participant’s bottom leaves the chair. Participants then walked three meters at their usual walking speed, turned around, and walked back to the chair and sat down. The stopwatch stopped when the participant’s bottom touched the chair once again. The average time (in seconds) of two performances was used as the result (28).

#### 2.5.3. Functional reach

The functional reach test was performed in the standing position. The participant stood against the wall with one arm positioned at 90° of forward flexion with a measuring tape placed at shoulder height. Participant was instructed to maintain their base of support with legs shoulder width apart while trying to reach forward as far as they could. The difference in length between the initial position of the tip of the middle finger (in cm) and the furthest position was considered the FR (29).

#### 2.5.4. Hand grip strength

To assess HGS, a Jamar™ hand dynamometer (Patterson Medical, United States) was used. Participants were seated with shoulders adducted, elbow flexed to 90 degrees, with the forearm and the wrist maintained in neutral position, while the hand gripping the dynamometer. The best result from the dominant hand was taken as the result for the present study (30).

### 2.6. Data analysis

Statistical analyses were conducted using the Statistical Package for Social Science for Windows, version 2,626 (IBM Corp., Armonk, N.Y., United States). The Independent t-test was used to generate descriptive characteristics for continuous variables while the Chi-squared test was used for categorical variables. The association between physical performance tests and the risk of sarcopenia was assessed using multiple logistic regressions. The physical performance test data were dichotomized based on the outcome value of the receiver operating characteristic curve (ROC) analysis. By categorizing participants as having or not having a risk of sarcopenia at 5 years, this approach creates a plot of sensitivity (true positive rate) vs. 1-specificity (false positive rate) at each test value. The test value with the highest sensitivity and specificity was then selected as a cut-off to classify whether the participant has the condition (31, 32). The SARC-F cumulative incidence was calculated by dividing new cases of SARC-F during the follow-up period by the number of participants at risk in the population at baseline. The incidence rate was obtained by dividing new cases of SARC-F by the total-person-time observed between the two assessments.

## 3. Results

### 3.1. Participants’ characteristics and incidence of SARC-F ≥ 4 in 5 years

Baseline and follow-up SARC-F scores were available for 747 participants. Participants’ baseline characteristics are presented in Table 1. Eighty-six (11.1%) fulfilled the SARC-F cut-off of four or more points and were classified as sarcopenic at baseline while 6.8% (n = 51) women from the total population were categorized as sarcopenia group, mean age (SD) 72.0 ± 6.98, *p* < 0.001. From the data analysis at follow-up, 632 (84.6%) participants were categorized as normal (SARC-F < 4), and 115 (15.4%) participants were in the sarcopenia category (SARC-F ≥ 4). The mean age for normal group participants was 67.5 ± 6.52 years, and 54.7% (n = 346) were women. The participant’s age range in the sarcopenic group was 70.8 ± 8.84 years, of which 67% (n = 77) were women. There were significant differences in body mass index (BMI), educational level, and presence of diabetes mellitus (DM) between groups (*p* < 0.001) (Table 1). The five-year incidence of possible sarcopenia according to SARC-F was 17 per 100 person-years.

**Table 1:** Characteristics of participants with SARC-F ≥ 4 and SARC-F < 4 at Baseline and 5 Years follow up (*N* = 747).

Characteristic	Baseline data		*p* value	5 years follow up		*p* value
	Normal (SARC-F < 4) *n* = 661	With SARC-F ≥ 4 (SARC-F ≥ 4) *n* = 86		Normal (SARC-F < 4) *n* = 632	With SARC-F ≥ 4 (SARC-F ≥ 4) *n* = 115	
Age, Year, Mean ± SD	67.52 ± 6.86	72.00 ± 6.98	**<0.001**	67.53 ± 6.52	70.77 ± 8.84	**0.001**
Gender, Female, *n* (%)	372 (56.3%)	51 (59.3%)	0.595	346 (54.7%)	77 (67.0%)	**0.015**
BMI, Mean *± SD*	24.62 ± 4.18	26.18 ± 5.00	**0.002**	24.56 ± 4.15	26.15 ± 4.90	**<0.001**
Marital status, single or no partner, *n* (%)	140 (21.3%)	25 (29.1%)	0.136	130 (20.6%)	35 (30.5%)	0.067
Educational level, primary or below, n (%)	113 (17.2%)	22 (25.6%)	0.085	89 (14.2%)	46 (40%)	**<0.001**
Comorbidities, *n* (%)						
Hypertension	267 (52.9%)	43 (72.9%)	**0.002**	260 (54.4%)	50 (58.1%)	0.520
Diabetes mellitus	147 (22.4%)	34 (40.5%)	**<0.001**	138 (21.4%)	48 (40.7%)	**0.001**
Heart disease	46 (4.3%)	11 (6.3%)	0.225	30 (6.3%)	9 (10.5%)	0.162
Stroke	7 (1.1%)	0 (0.0%)	0.429	9 (1.9%)	1 (1.2%)	0.256
CKD	14 (2.1%)	3 (3.6%)	0.301	14 (2.2%)	3 (2.6%)	0.806
COPD	2 (0.3%)	1 (1.2%)	0.303	2 (0.3%)	1 (0.9%)	0.393
High cholesterol	349 (53.1%)	55 (65.5%)	**0.021**	333 (53.2%)	71 (61.7)	0.091

Bold values of *p*-value <0.05 as indicated. CKD=Chronic Kidney Disease, COPD = Chronic obstructive pulmonary disease.

### 3.2. Physical performance among participants with and without SARC-F ≥ 4

**Figure 2:**
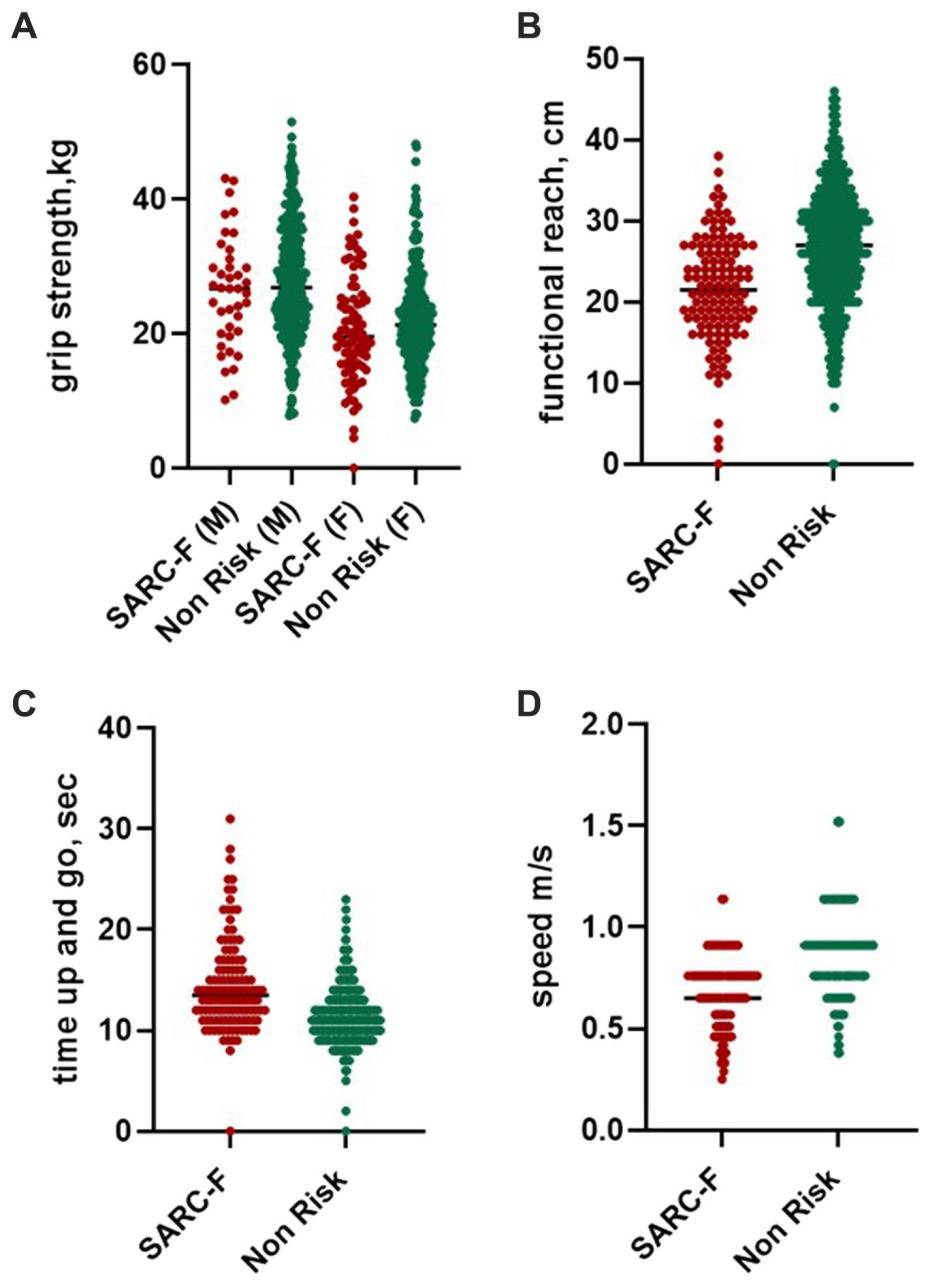
Physical performance score in non-risk participants and with a SARC-F score of 4 and above. Graph figure of physical performance in participants with SARC-F ≥ 4. SARC-F: S=Strength, A = Assistance in walking, R = Rising from a chair, C=Climbing stairs, F = Falls history.

Physical performance among participants with and without sarcopenia in the baseline and after five years of follow-up is summarized in Table 2. The TUG, GS, and FR were significantly correlated with the SARC-F scores (*p* < 0.001) at the baseline and follow-up. However, HGS in this study was not significant in predicting the risk of sarcopenia in both groups (Table 2; Figure 2).

**Table 2:** Physical Performance according to SARC-F.

Physical performance measures	Baseline (Mean *±* SD)		*p*-value	After 5 years follow-up (Mean *±* SD)		*p* value
	Normal	With SARC-F **≥** 4		Normal	With SARC-F ≥ 4	
Gait speed, m/s	0.84 ± 0.19	0.66 ± 0.16	**<0.001**	0.85 ± 0.18	0.67 ± 0.17	**<0.001**
Time up and go, sec	11.42 ± 2.86	15.13 ± 4.07	**<0.001**	11.37 ± 2.65	14.43 ± 4.59	**<0.001**
Hand grip strength, kg						
Male	27.19 ± 8.31	25.30 ± 8.05	0.066	27.65 ± 8.65	25.99 ± 7.89	0.263
Female	21.83 ± 7.10	20.66 ± 7.44	0.131	22.12 ± 6.93	20.59 ± 7.93	0.089
Functional reach, cm	26.28 ± 7.29	23.15 ± 7.52	**<0.001**	26.74 ± 7.17	21.39 ± 6.86	<0.001

Bold values of *p*-value <0.05 as indicated.

### 3.3. Cut off values for physical performance measures

The area under the ROC curve (AUC) was used to assess the performance of baseline GS, TUG, HGS, and FR in predicting probable sarcopenia in 5 years. The reference values set for GS were ≤ 0.7 m/s (72.9% sensitivity and 53% specificity). Meanwhile, ≥11.5 s was set for TUG (74.2% sensitivity and 57.2% specificity). We proposed a cut-off value of ≤22.5 cm for FR (73% sensitivity and 54.2% specificity). The cut-off values for male and female HGS proposed were < 22 kg and < 17 kg, respectively, (70% sensitivity for both genders, 26.7% specificity for male HGS, and 20.3% for female HGS) (Table 3).

**Table 3:** Rate ratio of SARC-F ≥ 4 at 5 years follow up among older people with poor physical performance measured using different type of test.

Physical Performance	Cut-off value	Sensitivity (%)	Specificity (%)	SARC-F ≥ 4, Rate Ratio, RR (95% CI)				
				Unadjusted model	Age-adjusted	Adjusted model 1	Adjusted model 2	Adjusted model 3
Gait Speed, m/s	0.70	79.2	53.0	**4.28 (2.85. 6.42)**	**3.65 (2.38–5.60)**	**2.69 (1.64–4.11)**	**2.45 (1.40–4.29)**	**2.29 (1.29–4.06)**
Time Up and Go, sec	11.5	74.2	57.2	**3.83 (2.48; 5.93)**	**3.33 (2.13; 5.21)**	**2.70 (1.69; 4.29)**	**1.97 (1.08; 3.25)**	**1.77 (1.00; 3.13)**
Functional reach, cm	22.5	73.2	54.2	**3.24 (2.17; 4.82)**	**2.85 (1.89; 4.29)**	**2.00 (1.28; 3.13)**	**1.76 (1.02; 3.05)**	**1.76 (1.01; 3.06)**
Hand grip strength, kg	M: <22	70.0	26.7	**1.71 (1.15; 2.56)**	**1.72 (1.14; 2.58)**	1.21 (0.77; 1.99)	1.07 (0.62; 1.84)	1.04 (0.60; 1.81)
	F: <17	70.0	20.3					

Bold fonts indicate significant at *p* < 0.05, CI = confidence interval.

Adjusted Model 1: Adjustment of age, Gender, Marital status, and Education level.

Adjusted Model 2: Adjustment made in Adjusted model 1 + Comorbidities (CKD, Emphysema/COPD, Diabetes, Hypertension, Cholesterol, Stroke).

Adjusted Model 3: Adjustment made in Adjusted model 2+ Baseline SARC-F.

### 3.4. Physical performance and sarcopenia

The association was significant for TUG, GS, and FR even after adjustment for baseline sarcopenia except for HGS. The rate Ratio (RR) of sarcopenia at five years follow-up among older people with poor physical performance was measured by an unadjusted model, adjusted Model 1 (model adjusted for age, gender, marital status, and educational level), adjusted Model 2 [adjustment made in adjusted model 1 + comorbidities (chronic kidney disease (CKD), emphysema/chronic objective pulmonary disease (COPD), diabetes, hypertension, cholesterol, stroke)] and adjusted Model 3 (adjustment made in adjusted model 2 + baseline SARC-F) as depicted in Table 3. The unadjusted model of GS was 4.28 (2.85; 6.42); TUG was 3.83 (2.48; 5.93) meanwhile FR 3.24 (2.17;4.82) and HGS 1.71 (1.15; 2.56). The HGS was the only parameter that was not significant after being adjusted with models 1, 2, and 3: 1.21 (0.77;1.99), 1.07 (0.62;1.84), and 1.04 (0.60;1.81) consecutively (Table 3).

## 4. Discussion

Aging is associated with balance impairment, and it is estimated that 13% of older people ages 65–69 and 46% of older adults ages 85 and above self-report having balance deficits (33). Lack of balance, unsteadiness while walking, and weak muscles are the main internal factors that increase the risk of falling in older adults (34). Older individuals with sarcopenia often experience weakened muscles, which can significantly impact their balance while walking. Early detection of sarcopenia is crucial in mitigating the progression of muscle loss and maintaining better health outcomes for older individuals. Implementing straightforward physical performance assessments in clinical practice becomes imperative to effectively prevent the occurrence of sarcopenia. Knowing the cut-off value of physical performance will empower health practitioners to establish targeted sarcopenia prevention programs effectively. Research conducted by Pepera et al. in 2021 revealed compelling evidence regarding the efficacy of a two-month multicomponent exercise training (MCEP) program in enhancing mobility among older adults. This comprehensive exercise regimen encompasses a combination of balance and muscle-strengthening exercises, strategically designed to amplify both balance performance and gait ability. Sarcopenia notably exerted a significant impact on these aspects in older adults.

This study identified the cut-off values for physical performance tests to predict the risk of sarcopenia in 5-years among individuals aged 55 years and over in Malaysia. The proposed cut-off value of gait speed was at ≤0.7 m/s, ≤11.5 s for TUG, and < 22.5 cm for FR. The cut-off of sex-specific hand grip strength for men was <22 kg women was <17 kg. Several studies have been conducted to predict probable sarcopenia utilizing SARC-F but were focused mainly on studying prevalence (35–39) and were not focused on the physical performance’s cut off values to predict sarcopenia risk in the Southeast Asian population.

The ability of TUG to predict sarcopenia has been mentioned in one study (11) with the cut-off set at 10.85 s (sensitivity of 67% and specificity of 88.7%). This result was slightly different from the present study which set the cut-off at ≤11.5 s (sensitivity of 74.2% and specificity of 57.2%). As physical performance is correlated with physiological and anthropometric measurements such as height and limb length that vary according to ethnicity, body morphology differences between European and Asian populations probably play a role in the dissimilarity of step length and walking speed resulting in the majority of the older Asian population having slower TUG (40).

In terms of gait speed, the cut-off point set in this study ≤0.7 m/s (sensitivity 79.2% and specificity 53%) was lower than the value set by the Asian Working Group of Sarcopenia (AWGS) which is <0.8 m/s (41). However, this cut-off was within a range value presented by Cawthon in his study which set the cut-off points in walking speed of 0.60 m/s and 0.75 m/s discriminated older adults with mobility limitation related to sarcopenia (42). Another study by Kang et al. (43) denoted healthy older adult walking speed was 1.23 ± 0.26 m/s, which is 0.08 m/s faster compared to sarcopenic people (1.15 ± 0.25 m/s, value of *p* <0.001).

In this study, the specificity for HGS cut-offs was quite low. This cut-off value of HGS was the lowest among the cut-off set by other working definition sarcopenia such as AWGS (<26 kg for men and < 18 kg for women) and EWGSOP (<30 kg for men and < 20 kg for women). The lack of association between HGS and sarcopenia defined using SARC-F at 5-year follow-up may be attributed to the limitation of SARC-F. A previous study has, however, suggested that SARC-F showed a high level of overlap with established definitions of sarcopenia of up to 54% with the IWGS definition (36). In addition, in the more recent study, it was suggested that SARC-F is better suited to rule out sarcopenia in case-finding. Since HGS just measures upper body strength, its value in determining whole-body strength is probably limited (44). However, according to research conducted by Laudisio et al. (45), a correlation exists between muscle strength, as quantified through handgrip strength, and the overall physical and mental well-being of older adults. Furthermore, enhancing muscle performance has the potential to contribute to an improved quality of life for this population.

### 4.1. Strength and limitation

This study represents the first longitudinal investigation into the relationship between physical performance measures (PPMs) including GS, TUG, HGS, and FR, and adverse health namely Sarcopenia in older Malaysian adults. However, this study lacked objective measurements of body composition and imaging (i.e., dual-energy X-ray absorptiometry and magnetic resonance imaging) to classify sarcopenia, which may have led to inaccuracies. Various imaging techniques, encompassing both qualitative and quantitative approaches, are utilized for evaluating muscle mass and body composition. These methods include computed tomography (CT), nuclear magnetic resonance (MRI), dual-energy X-ray absorptiometry (DXA), bioelectrical impedance analysis (BIA), and muscle ultrasound. CT and MRI are regarded as the benchmark due to their ability to provide accurate assessments of distinct body tissues, but a consensus regarding the specific cutoff values for defining sarcopenia remains absent (46). Secondly, the MELoR study targeted Malaysian adults living in an urban area which will limit the generalization to those living in rural areas whose occupations and lifestyles may be different. Future studies should include older people living in rural areas and compare their performance to evaluate whether geographical factors may affect the results. Finally, no physical assessment was conducted during the follow-up visit. Hence, we were not able to evaluate the influence of changes in physical performance on sarcopenia. Nevertheless, this study revealed the temporal associations between physical performance tests on five-year sarcopenia risk in older adults in Malaysia.

## 5. Conclusion

This study proposed the cut-off values for physical performance tests that may be useful for early detection of sarcopenia risk within the older Malaysian population. Future studies should seek to confirm our findings using more accurate sarcopenia measurements which should ideally include imaging modalities. In addition, the value of screening for five-year sarcopenia risk using physical performance tests should also be evaluated.

## Data availability statement

The raw data supporting the conclusions of this article will be made available by the authors, without undue reservation.

## Ethics statement

The studies involving humans were approved by the research ethics committee of the university of Malaya. The studies were conducted in accordance with the local legislation and institutional requirements. Written informed consent for participation was not required from the participants or the participants’ legal guardians/next of kin in accordance with the national legislation and institutional requirements.

## Author contributions

IM, SM, DKAS, and MPT conceived the study, contributed to the study design, obtained the funding for the study and were responsible for the conduct of the study. IM was involved in data collection. SM and MPT contributed to data analysis. All authors contributed to the writing of the manuscript and approved the final submitted version.
